# Links Between Burden of Common Infections and Cognitive Decline over More Than a Decade

**DOI:** 10.1093/geroni/igaf122.2295

**Published:** 2025-12-31

**Authors:** Chunyu Liu, David Sosnowski, Yiwei Yue, Alden Gross, Keenan Walker, Robert Yolken, Brion Maher, Adam Spira

**Affiliations:** Johns Hopkins Bloomberg School of Public Health, Baltimore, Maryland, United States; Johns Hopkins Bloomberg School of Public Health, Baltimore, Maryland, United States; Johns Hopkins Bloomberg School of Public Health, Baltimore, Maryland, United States; Johns Hopkins Bloomberg School of Public Health, Baltimore, Maryland, United States; National Institute on Aging, Baltimore, Maryland, United States; Johns Hopkins School of Medicine, Baltimore, Maryland, United States; Johns Hopkins Bloomberg School of Public Health, Baltimore, Maryland, United States; Johns Hopkins Bloomberg School of Public Health, Baltimore, Maryland, United States

## Abstract

A growing literature links common infections to dementia. We previously found, among participants in Wave 4 of the Baltimore Epidemiologic Catchment Area (ECA) Study that increased levels of antibodies to a greater number of common pathogens (Herpes Simplex Virus-1, Cytomegalovirus, Epstein-Barr virus, Varicella-Zoster virus, and Toxoplasma gondii) were cross sectionally associated with a greater number of errors on the Mini-Mental State Examination (MMSE). Here, we examined the association between infection burden and performance on the MMSE and verbal memory >10 years later in 291 ECA participants (mean age: 54.3±8.8 years; 63.4% women; 37.2% Black) who gave blood and completed cognitive testing at Wave 4 (2004-06) and repeated cognitive testing at Wave 5 (2016-22). We performed antibody tests for the aforementioned pathogens and summed the number of pathogens for which participants tested positive at Wave 4. After adjustment for age, sex, race, education, APOE genotype, and Wave 4 MMSE score, individuals with more infections at Wave 4 had a greater number of errors on the MMSE at Wave 5 (B = 0.134, p = 0.008). In similar analyses adjusting for Wave 4 word-list memory performance, more infections at Wave 4 were associated with poorer Wave 5 word-list delayed recall (B = –0.057, p = 0.041); associations with immediate recall (B = –0.042, p = 0.088) and recognition (B = –0.031, p = 0.080) did not reach significance. Results link infection burden to long-term global cognitive performance and memory. Studies investigating potential mechanisms linking infection burden to cognitive outcomes are needed.

